# Supplementary material for: Disproportionality analysis of drug-associated progressive multifocal leukoencephalopathy using spontaneous reports: A 20-year signal detection study based on the FAERS database
Source: PLoS One. 2026 Feb 3;21(2):e0341855. doi: 10.1371/journal.pone.0341855 (PMC12867222; doi:10.1371/journal.pone.0341855)
Supplement: S1 Table — (DOCX) [file pone.0341855.s003.docx]

| S1 Table List of 72 Drugs with Positive PML Signals in All Four Detection Algorithms, Ranked by Case Numbers | | | | | |
| --- | --- | --- | --- | --- | --- |
| DRUG | a | ROR (95% CI) | PRR (χ^2^) | EBGM(EBGM05) | IC(IC025) |
| NATALIZUMAB | 1848 | 38.08 (36.11-40.15) | 37.67 (49155.73) | 28.31 (27.08) | 4.82 (4.75) |
| RITUXIMAB | 1298 | 39.44 (37.13-41.9) | 38.96 (39423.66) | 32.16 (30.58) | 5.01 (4.92) |
| MYCOPHENOLIC ACID | 271 | 12.13 (10.74-13.7) | 12.08 (2652.3) | 11.67 (10.54) | 3.54 (3.37) |
| FINGOLIMOD | 249 | 8.01 (7.06-9.09) | 7.99 (1470.99) | 7.75 (6.97) | 2.95 (2.77) |
| METHOTREXATE | 186 | 3.76 (3.25-4.35) | 3.75 (366.28) | 3.68 (3.26) | 1.88 (1.67) |
| PREDNISOLONE | 145 | 11.38 (9.65-13.42) | 11.34 (1339.95) | 11.13 (9.7) | 3.48 (3.23) |
| DOXORUBICIN | 134 | 10.82 (9.11-12.84) | 10.77 (1166.72) | 10.59 (9.18) | 3.41 (3.15) |
| TACROLIMUS | 133 | 5.83 (4.91-6.92) | 5.82 (520.97) | 5.73 (4.96) | 2.52 (2.27) |
| CYCLOPHOSPHAMIDE | 120 | 10.53 (8.79-12.62) | 10.49 (1013.64) | 10.33 (8.88) | 3.37 (3.1) |
| PREDNISONE | 113 | 8.37 (6.95-10.08) | 8.35 (719.72) | 8.23 (7.05) | 3.04 (2.77) |
| BENDAMUSTINE | 101 | 28.03 (23.01-34.15) | 27.74 (2568.13) | 27.37 (23.2) | 4.77 (4.49) |
| CICLOSPORIN | 89 | 4.64 (3.77-5.72) | 4.64 (250.82) | 4.59 (3.85) | 2.2 (1.89) |
| DEXAMETHASONE | 89 | 5.51 (4.47-6.8) | 5.5 (324.01) | 5.45 (4.57) | 2.45 (2.14) |
| FLUDARABINE | 84 | 27.63 (22.26-34.3) | 27.35 (2108.87) | 27.05 (22.57) | 4.76 (4.44) |
| METHYLPREDNISOLONE | 77 | 7.96 (6.36-9.97) | 7.94 (462.31) | 7.87 (6.52) | 2.98 (2.65) |
| OCRELIZUMAB | 57 | 2.91 (2.24-3.78) | 2.91 (70.84) | 2.89 (2.33) | 1.53 (1.15) |
| BORTEZOMIB | 51 | 4.08 (3.1-5.37) | 4.07 (117.54) | 4.05 (3.22) | 2.02 (1.62) |
| HYDROXYCHLOROQUINE | 51 | 8.19 (6.22-10.79) | 8.17 (318.75) | 8.12 (6.45) | 3.02 (2.62) |
| MIRTAZAPINE | 49 | 6.83 (5.16-9.05) | 6.82 (241.76) | 6.78 (5.36) | 2.76 (2.35) |
| BRENTUXIMAB VEDOTIN | 47 | 17.06 (12.8-22.75) | 16.96 (701.43) | 16.85 (13.25) | 4.07 (3.66) |
| ALEMTUZUMAB | 44 | 10.12 (7.52-13.62) | 10.08 (358.04) | 10.03 (7.82) | 3.33 (2.89) |
| OBINUTUZUMAB | 42 | 14.06 (10.37-19.06) | 13.99 (503.84) | 13.91 (10.79) | 3.8 (3.36) |
| LAMIVUDINE | 37 | 9.18 (6.64-12.69) | 9.15 (267.3) | 9.11 (6.95) | 3.19 (2.72) |
| BUSULFAN | 35 | 14.67 (10.52-20.47) | 14.59 (441.25) | 14.53 (11.53 (11) | 3.86 (3.38) |
| DARATUMUMAB | 33 | 7.16 (5.09-10.09) | 7.15 (173.71) | 7.12 (5.34) | 2.83 (2.33) |
| AZATHIOPRINE | 32 | 13.96 (9.86-19.78) | 13.9 (381.4) | 13.84 (10.34) | 3.79 (3.29) |
| LOPINAVIR; RITONAVIR | 31 | 12.73 (8.94-18.14) | 12.68 (332.1) | 12.63 (9.39) | 3.66 (3.15) |
| OFATUMUMAB | 30 | 2.91 (2.04-4.17) | 2.91 (37.52) | 2.9 (2.15) | 1.54 (1.02) |
| RITONAVIR | 29 | 11.23 (7.79-16.19) | 11.19 (268.02) | 11.15 (8.21) | 3.48 (2.95) |
| ETOPOSIDE | 27 | 5.87 (02-8.57) | 5.86 (43) | 5.84 (26) | 2.55 (2) |
| MELPHALAN | 24 | 9.64 (6.45-14.4) | 9.6 (184.47) | 9.58 (6.84) | 3.26 (2.68) |
| VINCRISTINE | 24 | 8.26 (5.53-12.34) | 8.24 (152.18) | 8.21 (5.87) | 3.04 (2.46) |
| CARFILZOMIB | 22 | 4.18 (2.75-6.35) | 4.17 (52.91) | 4.16 (2.93) | 2.06 (1.45) |
| ZIDOVUDINE | 20 | 17.69 (11.39-27.48) | 17.58 (311.99) | 17.53 (12.13) | 4.13 (3.5) |
| AZACITIDINE | 19 | 2.99 (1.91-4.7) | 2.99 (25.14) | 2.99 (2.05) | 1.58 (0.93) |
| AXICABTAGENE CILOLEUCEL | 18 | 6.93 (4.36-11.02) | 6.92 (90.9) | 6.9 (4.68) | 2.79 (2.12) |
| LEFLUNOMIDE | 18 | 2.98 (1.88-4.74) | 2.98 (23.67) | 2.98 (2.02) | 1.57 (0.91) |
| NEVIRAPINE | 17 | 6.89 (4.28-11.1) | 6.88 (85.19) | 6.86 (4.61) | 2.78 (2.1) |
| STAVUDINE | 17 | 38.66 (23.94-62.45) | 38.11 (613.03) | 38.02 (25.45) | 5.25 (4.56) |
| ABACAVIR; LAMIVUDINE | 16 | 9.87 (6.04-16.14) | 9.84 (126.82) | 9.82 (6.51) | 3.3 (2.59) |
| BELATACEPT | 16 | 16.41 (10.03-26.84) | 16.31 (229.52) | 16.28 (10.78) | 4.02 (3.32) |
| CHLORAMBUCIL | 16 | 40.42 (24.65-66.26) | 39.81 (604.24) | 39.72 (26.27) | 5.31 (4.61) |
| DOLUTEGRAVIR | 16 | 7.84 (4.8-12.81) | 7.82 (94.96) | 7.8 (5.17) | 2.96 (2.26) |
| DARUNAVIR | 15 | 10.11 (6.09-16.8) | 10.08 (122.44) | 10.06 (6.58) | 3.33 (2.61) |
| IDELALISIB | 15 | 6.77 (4.08-11.24) | 6.76 (73.42) | 6.74 (4.41) | 2.75 (2.03) |
| IBRITUMOMAB TIUXETAN | 14 | 39.77 (23.44-67.45) | 39.18 (520.02) | 39.1 (25.13) | 5.29 (4.54) |
| EFALIZUMAB | 13 | 12.93 (7.49-22.31) | 12.87 (142.11) | 12.85 (8.14) | 3.68 (2.91) |
| EFAVIRENZ | 13 | 7.01 (4.06-12.08) | 6.99 (66.62) | 6.98 (4.42) | 2.8 (2.03) |
| BIKTARVY | 11 | 4.59 (2.54-8.3) | 4.59 (30.82) | 4.58 (2.79) | 2.2 (1.36) |
| ABACAVIR | 10 | 15.14 (8.13-28.2) | 15.05 (131.08) | 15.04 (8.93) | 3.91 (3.04) |
| CLADRIBINE | 10 | 4.45 (2.39-8.28) | 4.45 (26.68) | 4.44 (2.64) | 2.15 (1.28) |
| EPIRUBICIN | 10 | 3.92 (2.11-7.3) | 3.92 (21.72) | 3.92 (2.33) | 1.97 (1.1) |
| POLATUZUMAB VEDOTIN | 9 | 10.5 (2-19.25) | 9.97 (72.54) | 9.96 (5.75) | 3.32 (2.4) |
| ATG | 8 | 3.62 (1.81-7.24) | 3.62 (15.12) | 3.61 (2.02) | 1.85 (0.89) |
| ATAZANAVIR | 8 | 4.9 (2.45-9.81) | 4.89 (24.75) | 4.89 (2.73) | 2.29 (1.33) |
| THIOTEPA | 8 | 13.76 (6.87-27.57) | 13.69 (94.05) | 13.68 (7.64) | 3.77 (2.81) |
| TRIUMEQ | 7 | 4.17 (1.99-8.77) | 4.17 (16.86) | 4.17 (2.24) | 2.06 (1.04) |
| TECLISTAMAB | 7 | 10.28 (4.89-21.61) | 10.25 (58.37) | 10.24 (5.5) | 3.36 (2.33) |
| LAMIVUDINE; ZIDOVUDINE | 6 | 5.15 (2.31-11.47) | 5.14 (20.14 (20) | 5.14 (2.63) | 2.36 (1.27) |
| MITOXANTRONE | 6 | 8.47 (3.8-18.89) | 8.45 (39.39) | 8.44 (4.32) | 3.08 (1.98) |
| BASILIXIMAB | 5 | 7.83 (3.25-18.83) | 7.81 (29.66) | 7.8 (3.74) | 2.96 (1.78) |
| STRIBILD | 5 | 14.67 (6.09-35.35) | 14.59 (63.29) | 14.58 (6.99) | 3.87 (2.68) |
| GENVOYA | 5 | 5.28 (2.19-12.69) | 5.27 (17.28) | 5.26 (2.53) | 2.4 (1.22) |
| ELOTUZUMAB | 5 | 7.42 (3.08-17.85) | 7.4 (27.67) | 7.4 (3.55) | 2.89 (1.71) |
| EMTRICITABINE; TENOFOVIR | 5 | 6.75 (2.8-16.23) | 6.73 (24.39) | 6.73 (3.23) | 2.75 (1.57) |
| ENFUVIRTIDE | 5 | 10.13 (4.21-24.4) | 10.1 (40.98) | 10.09 (4.84) | 3.34 (2.15) |
| EPCORITAMAB | 4 | 10.63 (3.98-28.39) | 10.59 (34.73) | 10.59 (4.65) | 3.4 (2.11) |
| IDARUBICIN | 4 | 9.3 (37-24.03) | 8.97 (28.33) | 8.97 (3.94) | 3.16 (1.87) |
| MARAVIROC | 4 | 10.02 (3.75-26.75) | 9.98 (32.33) | 9.98 (4.39) | 3.32 (2.02) |
| CHLOROQUINE | 3 | 14.04 (4.51-43.68) | 13.97 (36.13) | 13.97 (5.4) | 3.8 (2.36) |
| DIDANOSINE | 3 | 7.43 (2.39-23.07) | 7.41 (16.63) | 7.41 (2.87) | 2.89 (1.44) |
| SAQUINAVIR | 3 | 18.49 (5.94-57.57) | 18.36 (49.25) | 18.35 (7.1) | 4.2 (2.75) |
