# Supplementary material for: Disproportionality analysis of drug-associated progressive multifocal leukoencephalopathy using spontaneous reports: A 20-year signal detection study based on the FAERS database
Source: PLoS One. 2026 Feb 3;21(2):e0341855. doi: 10.1371/journal.pone.0341855 (PMC12867222; doi:10.1371/journal.pone.0341855)
Supplement: Attachment — Submitted filename: Response to Reviewers.doc [file pone.0341855.s004.doc]

**Responses to Academic Editor’s Comments**

**Additional Editor Comments:**
The reviewers noted strengths of your work  but also several critical deficiencies. The main parts of the manuscript that need to be strengthened are discussion, limitations and conclusion. Pay very close attention to the recommendations of the reviewers as you revise.

**Comment 1:**

*“The main parts of the manuscript that need to be strengthened are discussion, limitations and conclusion.”*

**Response:**
We sincerely appreciate the editor’s valuable summary and guidance. In this revision, we have substantially strengthened the *Discussion*, *Limitations,* and *Conclusion* sections:

- The **Discussion section** has been reorganized to reduce excessive descriptions of drug mechanisms and to enhance comparison of ROR findings with previous literature, with an emphasis on the implications of the results for pharmacovigilance practice, regulatory decision‑making, and future research directions.
- The **Conclusion section** has been rewritten to condense the main findings, clarify that FAERS data reflect reporting trends rather than actual incidence, and propose new hypotheses regarding the monitoring of drug‑associated PML.
- The **Limitations section** now more explicitly addresses the inherent potential biases in the FAERS database, including reporting bias, diagnostic and regional limitations, and differences among clinical specialties in identifying PML cases.

**Comment 2:**

*“Please ensure that your manuscript meets PLOS ONE’s formatting requirements.”*

**Response:**
We have reformatted the entire manuscript by following the official PLOS ONE style templates and have carefully checked file naming conventions for all submissions, including the main manuscript, marked‑up revision file, and supplementary materials.

**Comment 3:**

*“PLOS requires an ORCID iD for the corresponding author.”*

**Response:**
The corresponding author’s ORCID iD has been verified and linked through the *Editorial Manager* system as required.

**Comment 4:**

*“The data repository noted in your Data Availability Statement does not qualify as an acceptable repository according to PLOS’s standards.”*

**Response:**
In response, we have now deposited the minimal dataset necessary for replication of our study findings in the public repository **Figshare** (DOI: <https://doi.org/10.6084/m9.figshare.30575225>) and have rewritten **Data Availability Statement**.
The uploaded dataset includes:

- The list of **7,244 report IDs** related to progressive multifocal leukoencephalopathy (PML) (as identified from the deduplicated *DEMO* table);
- The list of **229 unique drugs** included in our disproportionality analysis;
- Results from the **four signal detection methods** applied to these drugs.
  We have also updated the *Data Availability Statement* in the revised manuscript accordingly.

**Comment 5:**

*“Please pay close attention to the reviewers’ recommendations.”*

**Response:**
We have carefully addressed every point raised by both reviewers. Major revisions include reorganizing the discussion to strengthen scientific interpretation, refining data presentation and figure quality, clarifying methodological details, and expanding the explanation of data limitations. Comprehensive point‑by‑point responses are provided below.

**Responses to Reviewer’s Comments**

Reviewer #1:

**Comment 1:**

The manuscript presents an analysis of very interesting data that is worth adding to the published literature. The authors fulfill many recommendations for this article type (Fusaroli et al, Reporting of a Disproportionality Analysis for Drug Safety Signal Detection using Individual Case Safety Reports in PharmacoVigilance (READUS-PV): Development and Statement. Drug Saf 2024 May 7;47(6):575-584). Nevertheless, the manuscript itself has issues that warrant substantial rewriting and then re-review.

**Response:**
**We sincerely thank the reviewer for recognizing the value of our study. In response to your suggestions, we have thoroughly revised the manuscript to make it clearer and easier to understand. We have objectively presented our findings, clarified the conclusions, appropriately applied FAERS database data, distinguished between incidence and reporting rates, and removed any potentially confusing or misleading statements.**

**Comment 2:**

The authors analyze 20 years of data from the US FDA Adverse Events Reporting System (FAERS) to demonstrate the association of PML with medications. The description of the methods was reasonable; however there was not detailed information on how reports were excluded other than duplication. In figure 1, the arrows from the DEMO (after de-duplication), DRUG and REAC modules should all point together to the final dataset.

**Response:**
Thank you very much for this helpful comment. In response, we have **completely redrawn Figure 1** to better illustrate the relationship among the *DEMO*, *DRUG*, and *REAC* modules in the data‑integration process. The revised flowchart now clearly shows that all three modules were merged after the de‑duplication step to generate the final dataset for screening PML‑related reports.

Regarding the **exclusion criteria**, we have clarified in the *Methods* section that, apart from removing duplicate cases, we directly included all records containing the preferred term (*PT*) “progressive multifocal leukoencephalopathy (PML)” as identified in the REAC module. This revision improves the transparency of our data‑selection process and aligns the text with the updated Figure 1.

**Comment 3:**

The core analyses use 4 methods to quantify the level of association of medications with PML in an effort to avoid the bias of any one method (Reporting Odds Ratio, Proportional Reporting Ratio, Bayesian confidence propagation neural network [BCPNN], and multi-item gamma Poisson shrinker [MGPS]). All of these methods are on use by the US FDA and WHO and by researchers to analyze large data sets for drug and vaccine adverse events. They each have different characteristics. The authors chose to require that all 4 measures indicate an association as a conservative method to identify drug-PML associations. I cannot comment on the individual measures but was impressed with their requirement of agreement among the four. The authors report 72 drugs have significant associations with PML with the criterion that all 4 measures fulfill pre-defined levels. The four drugs with the strongest associations are descried in the text; details on the 4 measures for all 72 drugs are listed in Supplemental Table 1. It would be better to include Table 1, or at least part of the table with the highest impacted drugs, as part of the publication. Limited information regarding the demographics are provided (figures 2d and 2e).

**Response:**
We have incorporated the list of the 72 drugs with significant associations directly into the main text (previously contained in the Supplement). Given the space limitations, we highlighted the subset of drugs with the strongest signals in **Table 1**. Regarding demographic data, we have clarified in the manuscript that the FAERS dataset provides limited demographic dimensions, which inherently restricts the depth of such analyses.

**Comment 4:**

The graph of the time course of reports is interesting (figure 2a). The forest plot of RORs for the 72 medications (figure 3) is very interesting. The text mentions the strength of the ROR association for only a few medications; however no comment is made in the text about the widely varying strength of associations across the 72 medications nor is there an interpretation of the great differences in ROR confidence intervals.

**Response:**
We appreciate this insightful suggestion. We have revised the *Discussion* section to provide a detailed interpretation of the varying strengths of association and the wide confidence interval ranges observed for the ROR values across different drugs. We now discuss how these differences may reflect variable reporting frequencies, therapeutic contexts, and prior awareness of PML risk.

**Comment 5:**

Figure 5, graphically displaying the time course from drug initiation to PML diagnosis from the limited information in the FAERs case reports, is interesting but likely to be misleading because information on prior use and duration of other potentially relevant medications is not described and may not be available.

**Response:**

Thank you for this important observation. We acknowledge that while we found this aspect of the analysis interesting, we indeed overlooked its potential to mislead readers. We have made substantial revisions to address this concern. Specifically, we have added a comprehensive discussion of the limitations and inherent biases of the TTO analysis, ensuring that readers are fully informed of the constraints in interpreting these findings.

**Comment 6:**

The two main concerns with this manuscript are (1) the inherent bias of the data source and (2) the lack of a conclusion drawn from their analysis.
Regarding the data source, the authors make a partial acknowledgement of bias in the section on limitations. However, the text suggests that the incidence of events over 20 years is evident from FAERS, whereas this is just the incidence of reports. They cannot comment on population incidence of HIV associated or non-HIV associated PML from their data. The authors acknowledge the FAERS data are mostly from North America and Europe and comment that is likely due to geographic limitations on medication access; while that is true there are also likely geographic limitations on ability to diagnose PML (MRI scans, CSF JC virus nucleic acid amplification testing) and use of the US English language database.

**Response:**

We appreciate the reviewer’s comment and fully concur. We have substantially revised the **Limitations** section to clarify that our findings reflect FAERS reporting frequencies rather than true disease incidence. We explicitly acknowledge that FAERS data are subject to underreporting bias, differential reporting across specialties, and regional variation, and therefore cannot be used to estimate population-level incidence. **In addition, we have replaced all instances of the term “cases” with “reports” throughout the manuscript to avoid any potential misunderstanding by readers.** Following your guidance, these revisions significantly strengthen the scientific rigor and transparency of our manuscript.

**Comment 7:**
Another potential bias in the database is that PML may be more likely to be diagnosed by neurologists treating multiple sclerosis and hematologists treating lymphoma than rheumatologists or dermatologists using immunosuppressive therapies. Another potential bias in a database of spontaneous case reports is that there was a very early warning about the association of certain drugs. For example, early reports linked natalizumab with PML leading to the drug being unlicensed temporarily by the FDA then reinstated with a black box warning. Once this association was suspected, voluntary reporting of PML diagnosis among persons treated with this drug may have become more frequent.

**Response:**
This is an excellent and realistic point. We have expanded the ***Discussion*** section to discuss how professional specialization, disease focus, and prior regulatory alerts (e.g., the early natalizumab signal) may have influenced reporting frequency. These contextual factors have been acknowledged as potential sources of reporting bias in our interpretation.

**Comment 8:**
In the conclusion section, the authors write extensively about HIV associated with PML and IRIS. This is misplaced. HIV induced immunosuppression was a cause of PML before there were any antiretroviral medications. The discussion of HIV associated PML is not relevant except for the observation that reports of PML in FAERS were few and stable during the 20 years when reports of drug associated PML increased dramatically.

**Response:**
Thank you for this important clarification. We have removed the lengthy section on HIV and IRIS from the Discussion. A brief description of the clinical context of IRIS is now provided. In parallel, by referencing the 20-year trend in PML reports extracted from the FAERS database, we have made it clear that the number of reports of HIV-associated progressive multifocal leukoencephalopathy (PML) has remained relatively stable over time, in stark contrast to the marked increase in drug-associated PML reports. This sharp contrast is now explicitly highlighted in the revised manuscript.

**Comment 10:**

There is not much to say about steroids and risk of PML. A very few cases were reportedly associated with steroids which is remarkable when one considers how widespread is the use of therapeutic systemic corticosteroids. Importantly, I do not find a new conclusion based on the data. Reports of drug associated PML have increased over 20 years, some of the PML patients had immunosuppressive disorders and almost all the rest received medications that were immunosuppressive. I did not find where the authors offered a new hypothesis derived from their analysis; that would be appropriate to justify publishing this manuscript.

**Response:**
we have revised the Conclusion to emphasize that drug‑induced PML has become a key focus of pharmacovigilance. This study systematically characterizes high‑risk populations and disease patterns (including multiple sclerosis, lymphoma, autoimmune diseases, and organ transplantation), reveals that distinct immunosuppressive mechanisms—lymphocyte depletion and interference with DNA synthesis—converge on impaired immune surveillance pathways leading to PML, and identifies an extended latency window (49–1343 days) that challenges traditional short‑term monitoring strategies. We further propose that practical biomarkers such as CD4⁺ cell counts may facilitate risk stratification in clinical settings.

Reviewer #2:

**Comment 1:**

The paper presents data on an adverse drug database, linking the PML infection to the use of immunosuppressant drugs and describing demographic and drug data of these adverse drug reactions. Although it is a well-conducted study with a large dataset, the manuscript lacks a robust discussion that can demonstrate the importance and relevance of its findings.

**Response:**
We have thoroughly rewritten the Discussion to better highlight the importance and relevance of our findings. The revised text now focuses on the implications for pharmacovigilance practice, clinical decision‑making, and public‑health policy, and includes clear comparisons with previous studies addressing similar topics.

**Comment 2:**

Furthermore, the figures presented in the manuscript are of poor quality, making it difficult to read their captions and fully understand them.

**Response:**
All figures (Figures 1–5) have been fully revised and re‑exported at high resolution (300 dpi). Following PLOS ONE’s official requirements, we used the Art Analysis (PageMajik) online tool provided by the journal to verify image resolution, font embedding, and color compliance. All figures passed the quality check and now comply with PLOS ONE’s technical specifications. Please note that the figures may appear slightly compressed in the generated PDF due to the system’s automatic optimization process; we kindly ask you to refer to the original image files for full‑resolution quality.

**Comment 3:**
The methods used to analyze the adverse drug reactions should be fully described in the manuscript, and their use discussed as a way to provide clinical and policy evidence. I believe the same applies to the validation methods cited in lines 93-94 for the case identification. It is important to ensure reproducibility of the research.

**Response:**
We appreciate the reviewer’s valuable comment regarding the need for clear methodological description and reproducibility. In the revised manuscript, we have clarified the references to the established methodologies cited in lines 93–94, which are widely recognized in pharmacovigilance research and validated in prior publications. These references provide the detailed algorithms and validation procedures for adverse event case identification and signal detection.

To further improve transparency, we have refined the workflow figure to illustrate the screening and exclusion process more specifically and deposited the minimal dataset required to replicate our findings in the public repository **Figshare** (DOI: [10.6084/m9.figshare.30575225](https://doi.org/10.6084/m9.figshare.30575225)). The Data Availability Statement has also been updated accordingly. These additions enhance the accessibility and reproducibility of the study without redundant repetition of well‑established methods.

**Comment 4:**
The discussion section focuses a lot on the drugs and their mechanisms of action. Instead, it should focus on the findings' implications for practice, public health policy and research. It would also be important to relate and contrast it with other research in the field that either uses similar methods, drugs, or problems to contextualize your research in the scientific field. How does it advance the research in the theme? How is it similar or different from the ones already published? How can these differences be explained? What are the manuscript's main contributions?

**Response:**
We have fully revised the ***Discussion*** section as suggested. The updated version now focuses on:

- The implications of our results for clinical decision‑making and pharmacovigilance practice;
- How our findings complement and differ from previously published studies using FAERS and other spontaneous‑reporting databases;
- The potential public‑health relevance of emerging drug‑related PML signals; and
- The contribution of this study to advancing the methodological understanding of signal detection in large‑scale pharmacovigilance datasets.

**Comment 5:**

As minor corrections, I suggest reviewing the points listed above:

1. Line 59: Correct reference format
2. Lines 60-63: A reference should be included to support it.
3. Lines 86: The link should be included as a reference.
4. The quality of the figures is not very good. It is not possible to read the captions in the figures, for instance, in 2a, to identify which color represents HIV or non-HIV PML infection. Neither is it possible to read the years in x. The same applies to Figures 2b and 2d; it is not possible to read what is in the graphs. In Figure 3, it is not possible to read the names of the drugs, even when using zoom.
5. I do not understand why there are two types of graphs for the same numbers in Figure 4.
6. Lines 227-228: Your data do not support this statement: “and that the use of immunomodulatory drugs is more prevalent”. This could be a hypothesis and along with a reference on drug use, that may indicate it.
7. Lines 231-233: It is important to include a reference that expresses that autoimmune diseases are more prevalent in women. The same applies to the sentence in lines 234-236.
8. Lines 377-378: “for drugs with unclear mechanisms that showed positive signals in our analysis but lack established causal relationships”: which drugs fall into this? Shouldn´t it be stated and discussed along with the results?

**Response**

1. The reference format at the original line 59 has been corrected, and two additional relevant references have been added (now appearing at line 58 in the revised manuscript).
2. Supporting references 15–17 have been added to this statement (line 60 in the revised manuscript).
3. We included the online source as a proper reference for line 86(reference 21).
4. All figures have been updated using the PLOS ONE recommended image preparation tool (https://ngplosjournals.pagemajik.ai/artanalysis). The revised figures now meet the journal’s resolution and format standards. We also ensured that all captions and axis labels are fully legible in the original high‑resolution files.We kindly suggest that the reviewer view the figures using the original high‑resolution versions provided with the submission to assess the improved readability of labels, captions, and color coding (e.g., HIV vs. non‑HIV PML in Figure 2 and drug names in Figure 3).
5. Two types of graphs were intentionally combined in Figure 4 to convey complementary information. The bar chart presents the absolute number of PML‑associated drugs in each pharmacological category, while the sunburst chart illustrates the proportional composition within the largest category—immunomodulatory drugs—showing its three major subgroups (drugs targeting lymphoid B and T cells, broad‑spectrum immunosuppressants, and small‑molecule immunomodulators).
6. We agree that our previous wording was ambiguous. Figure 2C represents the distribution of reported PML cases rather than the prevalence of drug use. We have revised the wording in this section and throughout the manuscript accordingly.To provide context, recent pharmacovigilance data show that PML reports are predominantly concentrated in **Europe and North America**, likely reflecting both stronger pharmacovigilance infrastructures and the widespread use of immunomodulatory agents in these regions (Jiang et al., *Front Pharmacol* 2022).
7. In the revised version (lines 276–279), we have added supporting references 33–35 to substantiate the statement that autoimmune diseases are more prevalent in women, in line with previous epidemiological evidence.
8. Following the reviewer’s suggestion to streamline the discussion of PML mechanisms, we have condensed this section in the revised manuscript. Accordingly, the sentence concerning drugs with unclear mechanisms has been removed, and the related references are no longer listed.

Thanks again for help!

Yours,

LJSG
