# Supplementary material for: Disproportionality analysis of drug-associated progressive multifocal leukoencephalopathy using spontaneous reports: A 20-year signal detection study based on the FAERS database
Source: PLoS One. 2026 Feb 3;21(2):e0341855. doi: 10.1371/journal.pone.0341855 (PMC12867222; doi:10.1371/journal.pone.0341855)
Supplement: Attachment — Submitted filename: response to reviewers R2.docx [file pone.0341855.s005.docx]

**Reviewer #1**

Comment 1) The manuscript does not clearly and succinctly state the specific contribution to medical knowledge.

**Response:** Thank you for this helpful suggestion. We have revised both the Introduction and the opening paragraph of the Discussion to consistently and explicitly state the study’s key contributions. Specifically, we clarify that our analysis uses FAERS data from 2004–2024 (updated to 2024Q4), provides an all-drug, indication-agnostic panoramic screen of drug–PML reporting, and applies four complementary disproportionality algorithms (ROR, PRR, BCPNN, and MGPS) with a concordance-only signal definition to improve specificity, yielding an updated and conservative signal inventory for prioritizing further validation. **(Introduction, last paragraph).**

Comment 2) Confounding by indication: difficulty separating drug–PML association from underlying conditions (esp. HIV, lymphoma).

**Response:** Thank you for pointing out this key interpretive issue. A major feature of our study is the systematic identification of signals for all drugs reported with PML over an extended FAERS time window. We acknowledge that these signals inevitably conflate potential drug–PML associations with the background PML risk attributable to the underlying indications (particularly HIV and lymphoma), as well as drugs implicated in PML pathogenesis and drugs used to treat PML. Given the limited clinical detail and incompleteness of FAERS reports, we cannot reliably disentangle these effects at the individual level. We have therefore added an explicit statement in the last paragraph of the Discussion to highlight this limitation and to frame our findings as hypothesis-generating rather than causal **(Discussion, 3. Pathogenic Mechanisms and Risk Features of Key Drugs).**Comment 3) Explain the four disproportionality methods and why requiring concordance is beneficial.

**Response:** We have revised the beginning of the “Signal detection methods” description to briefly clarify the conceptual differences among the four algorithms and the rationale for combining them. Specifically, we added 2–3 sentences to explain that ROR/PRR are frequentist disproportionality measures based on 2*2 contingency tables and may be sensitive to sparse counts, whereas BCPNN/MGPS are Bayesian shrinkage approaches that stabilize estimates for rare drug–event pairs. We also clarified that we required concordant signals across all four methods to prioritize specificity and reduce spurious findings, while acknowledging a potential loss of sensitivity. **(Materials and methods, 1. Signal Detection Methods)**

Comment 4) Sex differences: avoid inferring sex-specific susceptibility; differences likely reflect epidemiology of underlying conditions.

**Response:** We agree. We revised the Discussion to avoid inferring sex-specific susceptibility. We now interpret the observed sex distribution as more likely reflecting the epidemiology of underlying diseases and treatment patterns (e.g., HIV predominance among men; autoimmune diseases predominance among women), and we explicitly note that FAERS disproportionality signals do not permit causal inference regarding sex-specific risk. **(Discussion, 1.Trends in PML Epidemiology and Changes in Population Characteristics, last paragraph)Comment 5)** CD4 statements are too strong / unsupported; not applicable to rituximab/natalizumab contexts.

**Response:** Thank you for raising this important point. We fully agree that rituximab does not directly target CD4+ T lymphocytes and that peripheral CD4+ T lymphocyte counts may increase during natalizumab therapy.We would like to clarify that our statement regarding "CD4+ T lymphocyte measurements may help assess the risk of drug-associated PML" was not intended to present CD4+ T lymphocytes as a direct "risk biomarker" or as causal evidence. Rather, our rationale is based on the core pathogenesis of PML—reactivation of JCV in the context of immunosuppression or immune reconstitution. Within this framework, the number and functional status of CD4+ T lymphocytes can, to some extent, indirectly reflect overall immune competence and therefore serve as a practical "barometer" of immune status. Even when certain drugs do not act directly on CD4 (e.g., rituximab primarily depletes B cells), CD4-related immune dysfunction may still occur due to the underlying disease, immune cell interactions, or sustained immunosuppression. Therefore, from this perspective, CD4+ T lymphocyte-centered assessments—including counts, functional status evaluation, and specific subset characteristics—may serve as part of supportive risk stratification, rather than being used in isolation to draw conclusions. Your suggestions have helped us better refine our statement.

Regarding the apparent paradox of "increased CD4+ T lymphocyte counts" with natalizumab, we carefully reviewed the corresponding article you provided and incorporated its findings into our manuscript. However, we noted that the article suggests the increase in peripheral blood CD4+ T lymphocyte counts may more likely reflect the drug's pharmacological mechanism—namely, its inhibition of lymphocyte migration. The authors mentioned twice in the article that the study cannot answer whether discontinuing natalizumab would reduce PML incidence, and that the study was not designed to assess the impact of natalizumab dosing interruption on PML risk. Nevertheless, based on the phenomenon of "increased CD4+ T lymphocyte counts" during natalizumab therapy that you highlighted, we have further enriched the depth of our literature support by introducing CD4+ T lymphocyte functional status/subset count characteristics, thereby better completing the evidence chain.

Regarding rituximab, although it directly acts on B cells, previous studies have found that CD4+ T lymphocytes decrease during rituximab maintenance therapy, and CD4+ T lymphocyte counts show an upward trend when maintenance is discontinued, suggesting a synergistic effect between B/T cells. We have also cited the corresponding literature to support this argument.

In summary, we again thank the reviewer for helping us clarify the appropriate scope of this statement and for prompting us to present this point and its limitations more clearly in the Discussion section.In response to the above comments, we have made revisions in the following section **(Discussion, 3. Pathogenic Mechanisms and Risk Features of Key Drugs, second paragraph)**.

、

1. Focosi D, Tuccori M, Maggi F. Progressive multifocal leukoencephalopathy and anti-CD20 monoclonal antibodies: What do we know after 20 years of rituximab? **Rev Med Virol.** 2019;29(6):e2077. doi:10.1002/rmv.2077. PMID:31369199.
2. Berkovich R, Togasaki DM, Cen SY, Steinman L. CD4 cell response to interval therapy with natalizumab. **Ann Clin Transl Neurol.** 2015;2(5):570-574. doi:10.1002/acn3.190. PMID:26000328.
3. Haghikia A, Perrech M, Pula B, Ruhrmann S, Potthoff A, Brockmeyer NH, et al. Functional energetics of CD4+ cellular immunity in monoclonal antibody-associated progressive multifocal leukoencephalopathy in autoimmune disorders. **PLoS One.** 2011;6(4):e18506. doi:10.1371/journal.pone.0018506. PMID:21533133.
4. Dubois E, Ruschil C, Bischof F. Low frequencies of central memory CD4 T cells in progressive multifocal leukoencephalopathy. **Neurol Neuroimmunol Neuroinflamm.** 2015;2(6):e177. doi:10.1212/NXI.0000000000000177. PMID:26568972.
5. Lavielle M, Mulleman D, Goupille P, et al. Repeated decrease of CD4+ T-cell counts in patients with rheumatoid arthritis over multiple cycles of rituximab treatment. **Arthritis Res Ther.** 2016;18(1):253.
6. Piantoni S, Scarsi M, Tincani A, Airò P. Circulating CD4+ T-cell number decreases in rheumatoid patients with clinical response to rituximab. **Rheumatol Int.** 2015;35(9):1571-1573.
7. Yutaka T, Ito S, Ohigashi H, et al. Sustained CD4 and CD8 lymphopenia after rituximab maintenance therapy following bendamustine and rituximab combination therapy for lymphoma. **Leuk Lymphoma.** 2015;56(11):3216-3218.

**Response to Reviewer #2**

Dear Reviewer,

Thank you very much for your careful re-evaluation of our manuscript and for your constructive suggestions. We have revised the manuscript accordingly. Point-by-point responses are provided below.

**Comment 1)** Figure 2 Legends: The legends for Figure 2 are incorrect (2c vs 2d).

**Response:** Thank you for pointing this out. We corrected the mismatch between the Figure 2c and Figure 2d legends. The revised legend now accurately describes Figure 2c as geographic distribution reporting and Figure 2d as reporter/professional category reporting, and we verified that all in-text citations to Figure 2 subpanels are consistent. **(Figure 2 legend; Results, Characteristics of Reported PML Reports in FAERS)**

**Comment 2)** Missing Citations: The second paragraph of the Discussion, Section 3 (lines 323–330), requires a reference citation.

**Response:** Thank you for noting this. We added supporting citations to the mechanistic statements in this paragraph, including references [1] and [12] for the link between immunosuppression and PML/JCV reactivation, and a review by Cain & Cidlowski [46] for the immunoregulatory mechanisms of glucocorticoids. **(Discussion, 3. Pathogenic Mechanisms and Risk Features of Key Drugs)**

1.Tan CS, Koralnik IJ. Progressive multifocal leukoencephalopathy and other disorders caused by JC virus: clinical features and pathogenesis. Lancet Neurol. 2010;9(4):425-437. doi:10.1016/S1474-4422(10)70040-5. PMID:20298966.

12.Cortese I, Reich DS, Nath A. Progressive multifocal leukoencephalopathy and the spectrum of JC virus-related disease. Nat Rev Neurol. 2021;17(1):37-51. doi:10.1038/s41582-020-00427-y. PMID:33219338.

46.Cain DW, Cidlowski JA. Immune regulation by glucocorticoids. Nat Rev Immunol. 2017;17(4):233-247. doi:10.1038/nri.2017.1. PMID:28192415.

**Comment 3)** Punctuation Review: The third paragraph of the Conclusion section and the Limitations section need punctuation corrections.

Response: Thank you. We proofread the **Conclusion** and **Limitations** sections and corrected punctuation/typographical issues (e.g., missing spaces after periods, redundant punctuation, and sentence formatting). We also improved sentence readability in the **Limitations section** without changing the meaning.
